# Racial and ethnic disparities of sudden unexpected infant death in large US cities: a descriptive epidemiological study

**DOI:** 10.1186/s40621-022-00377-7

**Published:** 2022-03-25

**Authors:** Brett T. Boyer, Gina S. Lowell, Douglas R. Roehler, Kyran P. Quinlan

**Affiliations:** 1 Rush Medical College, Chicago, IL USA; 2 Department of Pediatrics, Rush University Medical Center, 1725 W Harrison St Suite 710, Chicago, IL 60612 USA

**Keywords:** SUID, Race and ethnicity, US cities

## Abstract

**Background:**

Sudden unexpected infant death (SUID) accounts for ~ 3400 deaths per year in the USA, and minimal progress has been made in reducing SUID over the past two decades. SUID is the sudden death of an infant that has occurred as a result of accidental suffocation in a sleeping environment, SIDS (sudden infant death syndrome), or from an unknown cause of death. Nationally, non-Hispanic Black (NHB) infants have twice the risk of SUID compared to non-Hispanic White (NHW) infants. In Chicago, this disparity is greatly magnified. To explore whether this disparity is similarly seen in other large cities, we analyzed SUIDs by race and ethnicity for a seven-year period from the 10 most populous US cities. SUID case counts by race and ethnicity were obtained for 2011–2017 from the 10 most populous US cities based on 2010 census data. For each city, we calculated average annual SUID rates (per 1000 live births) by race and ethnicity, allowing calculation of disparity rate ratios.

**Findings:**

Nationally, from 2011 through 2017, there were 0.891 SUIDs per 1000 live births, with a rate of 0.847 for NHWs, 1.795 for NHBs, and 0.522 for Hispanics. In most study cities, the NHB and Hispanic SUID rates were higher than the corresponding national rate. Hispanic SUID rates were higher than NHW rates in 9 of the 10 largest cities. In every study city, the NHW SUID rate was lower than the national NHW rate. In Chicago, NHB infants had a SUID rate 12.735 times that of NHW infants.

**Conclusion:**

With few exceptions, the 10 largest US cities had higher NHB and Hispanic SUID rates, but lower NHW SUID rates, compared to the corresponding rates at the national level. Unlike the national pattern, Hispanic SUID rates were higher than NHW rates in 9 of the 10 largest cities. Prevention is currently hampered by the lack of detailed, accurate, and timely information regarding the circumstances of these tragic deaths. A national SUID surveillance system would allow greater understanding of the factors that lead to this disproportionately distributed and enduring cause of infant death.

## Introduction

Sudden unexpected infant death (SUID) accounts for ~ 3400 deaths per year in the USA, and while the Back to Sleep campaign reduced SUID in the USA in the 1990s, minimal progress has been made in reducing SUID over the past two decades (Centers for Disease Control and Prevention 2021). A SUID is the sudden death of an infant younger than one year of age that has occurred as a result of accidental suffocation in a sleeping environment, SIDS (sudden infant death syndrome), or from an unknown cause of death. Nationally, non-Hispanic Black (NHB) infants have twice the risk and Hispanic infants have a little over half the risk of SUID compared to non-Hispanic White (NHW) infants (Parks et al. 2017). However, in Cook County, Illinois, which includes the city of Chicago, SUIDs are 13 times higher among NHB infants and over 2 times higher among Hispanic infants compared to NHW infants (Roehler and Quinlan 2018). To explore whether other large US cities have racial and ethnic disparities similar to that of Chicago, we analyzed SUID data for the 10 most populous US cities. We chose the 10 most populous cities because we observed Chicago had a disproportionate incidence of Black and Hispanic infants dying of SUID when compared to their white counterparts. If SUIDs are similarly increased for Black and Hispanic infants in other large US cities, further inquiry, novel partnerships, and focused prevention strategies could be employed.

## Methods

SUID case counts by race and ethnicity were obtained for 2011–2017 from the 10 most populous US cities based on 2010 census data (by population size: New York, Los Angeles, Chicago, Houston, Phoenix, Philadelphia, San Antonio, San Diego, Dallas, San Jose). The most recent year of data available among all study cities was 2017. SUIDs were defined by International Classification of Diseases-Tenth Revision codes R95 (Sudden infant death syndrome), R99 (Ill-defined and unknown cause of mortality) and W75 (Accidental suffocation and strangulation in bed) (Kim et al. 2012). Data include counts of SUIDs and live births of city residents by race and ethnicity for 2011–2017. For each city, we calculated average annual SUID rates (per 1000 live births) by race and ethnicity, allowing calculation of disparity rate ratios. While SUID rates are high among American Indian/Alaska Native (AI/AN) populations, the AI/AN population is relatively small in large US cities and were not included in this analysis. California cities suppressed yearly SUID counts for individual race/ethnicity groups for years with < 10 cases. In these cases, we calculated upper and lower values of SUID rates and disparity rate ratios. All city data were obtained from individuals working in their respective health departments and rates calculated from that data. The national rate was calculated using the live births by race and ethnicity in the linked birth–death infant mortality dataset from the National Vital Statistics System (United States Department of Health and Human Services (US DHHS) 2021).

## Results

**Fig. 1:**
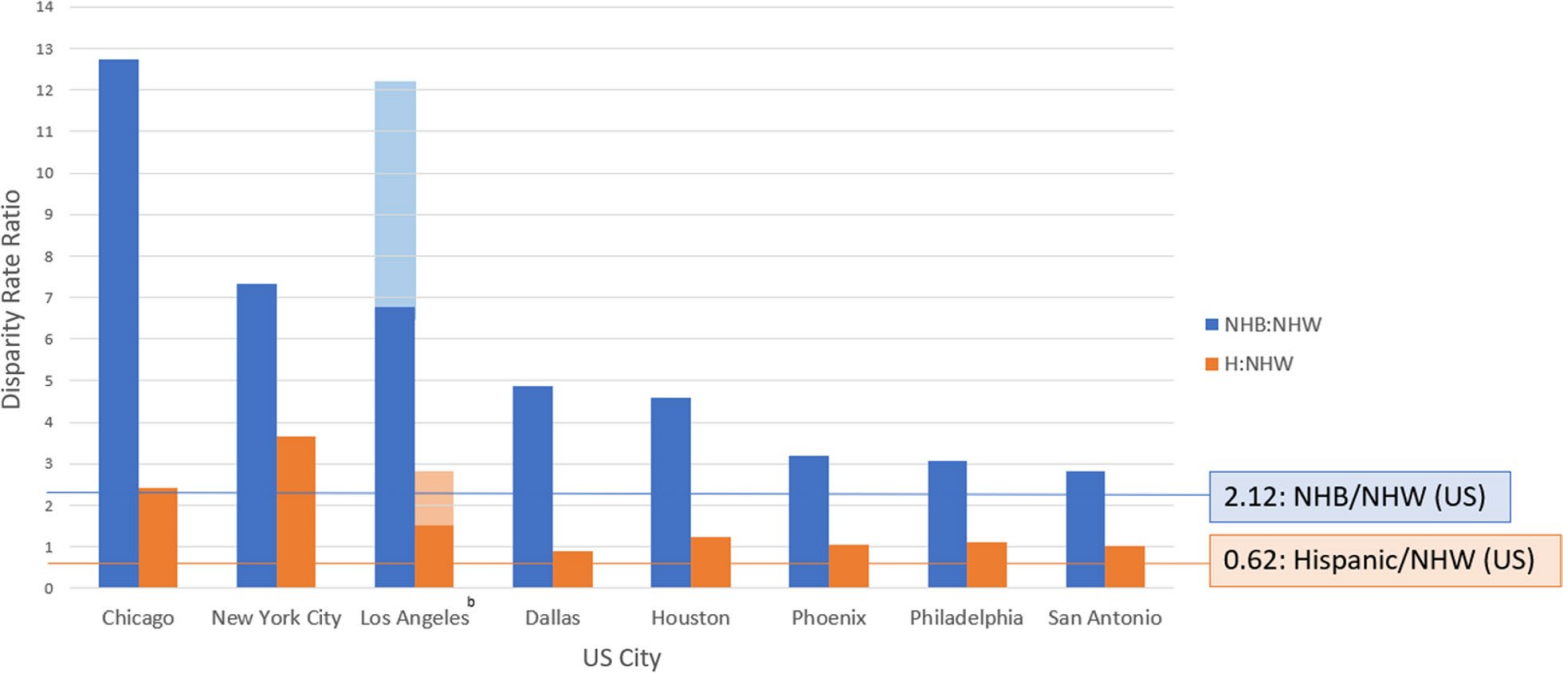
Sudden unexpected infant death disparity rate ratios by race and ethnicity for 8 large US Cities, 2011–2017. ^a^Two California cities (San Jose and San Diego) could not be included in the figure because some counts were suppressed due to small cell sizes. ^b^For Los Angeles, the Non-Hispanic Black:Non-Hispanic White ratio is shown as a minimum (dark blue vertical bar) and maximum (light blue vertical bar), and the Hispanic:Non-Hispanic White ratio is shown as a minimum (dark orange vertical bar) and maximum (light orange vertical bar) because some cells for Non-Hispanic White SUIDs were suppressed due to small cell sizes

Nationally, from 2011 through 2017, there were 0.891 SUIDs per 1000 live births, with a rate of 0.847 for NHWs, 1.795 for NHBs, and 0.522 for Hispanics (Table 1). In every study city, the NHW SUID rate was lower than the national NHW rate. In nearly every study city, the NHB and Hispanic SUID rates were higher than the corresponding national rate. Chicago had the highest NHB SUID rate (2.878), and Philadelphia had the highest Hispanic SUID rate (0.891). New York had the lowest SUID rate for all three groups: NHW (0.030), NHB (0.220), and Hispanic (0.110). In Chicago, NHB infants had a SUID rate 12.735 times that of NHW infants—the largest NHB:NHW disparity among study cities (Fig. 1).

**Table 1:** Sudden unexpected infant death (SUID) average annual rates per 1000 live births by race and ethnicity in the USA overall and for the 10 most populous cities, 2011–2017

	Non-Hispanic White	Non-Hispanic Black	Hispanic
New York	0.030	0.220	0.110
Los Angeles	0.162–0.292^a^	1.967	0.446
Chicago	0.226	2.878	0.550
Houston	0.476	2.185	0.597
Phoenix	0.527	1.693	0.550
Philadelphia	0.811	2.484	0.891
San Antonio	0.829	2.339	0.856
San Diego	0.126–0.226^a^	0.686–1.236 ^a^	0.626
Dallas	0.563	2.739	0.509
San Jose	0.060–0.239 ^a^	0.475–1.898 ^a^	0.409
USA	0.847	1.795	0.522

^a^For Los Angeles, San Diego and San Jose, the number of SUIDs was suppressed for some categories due to small cell size. In these situations, the range of possible SUID rates was calculated

## Discussion

With few exceptions, the 10 largest US cities had higher NHB and Hispanic SUID rates, but lower NHW SUID rates, compared to the corresponding rates at the national level. This pattern drove disparities by race and ethnicity in these cities. The NHB:NHW SUID disparity was greatest in Chicago where the NHB SUID rate was nearly 13 times that of NHW infants. Unlike the national pattern, Hispanic SUID rates were higher than NHW rates in 9 of the 10 largest cities. The Hispanic:NHW SUID disparity was greatest in New York City, which had the lowest SUID rates for all three groups. Why the largest US cities have higher NHB and Hispanic SUID rates, but lower NHW SUID rates compared to the nation deserves further inquiry. Demonstrating significant race and ethnic disparities in SUID exist not only in Chicago, but across multiple large US cities raise questions regarding the reasons why these disparities exist. Chicago’s infant mortality rates (IMR) are known to be concentrated in community areas experiencing racial segregation and economic marginalization (Bishop-Royse et al. 2021). Such communities experience a concentration of multiple drivers of health inequities, including access to health care, affordable and safe housing, and economic security. Collaborating with communities to explore how such drivers intersect with infant sleep circumstances is needed to understand how to develop meaningful and effective prevention. Limitations of the study include the use of individual city databases to obtain the data. While the conventional definition of SUID based on ICD-10 codes (R95, R99, and W75) is widely accepted, we did not have direct access to data for each city. Since this work only includes the 10 most populous US cities, further study is needed to explore how US SUID disparities vary with a larger range of cities and with urban/rural areas more generally. The CDC’s SUID case registry provides rich detail on circumstances (e.g., environment, sleep position, location) for about a third of all US SUIDs (Centers for Disease Control and Prevention’s 2021). Expanding this system to collect detailed data on all US SUIDs would be invaluable to better understand both upstream and modifiable factors that lead to these disparities, informing prevention work to better address them.

## Data Availability

The datasets generated and/or analyzed during the current study are available in the CDC Wonder repository, or available by request from individual cities public health departments or the corresponding author.

## Abbreviations

SUID: Sudden unexpected infant deaths
NHB: Non-Hispanic Blacks
NHW: Non-Hispanic Whites
IMR: Infant mortality rate
